# PPAR**γ** Signaling Mediates the Evolution, Development, Homeostasis, and Repair of the Lung

**DOI:** 10.1155/2012/289867

**Published:** 2012-06-26

**Authors:** Virender K. Rehan, John S. Torday

**Affiliations:** Department of Pediatrics, Los Angeles Biomedical Research Institute at Harbor-UCLA Medical Center, David Geffen School of Medicine, University of California at Los Angeles, Torrance, CA 90502, USA

## Abstract

Epithelial-mesenchymal interactions mediated by soluble growth factors determine the evolution of vertebrate lung physiology, including development, homeostasis, and repair. The final common pathway for all of these positively adaptive properties of the lung is the expression of epithelial parathyroid-hormone-related protein, and its binding to its receptor on the mesenchyme, inducing PPAR*γ* expression by lipofibroblasts. Lipofibroblasts then produce leptin, which binds to alveolar type II cells, stimulating their production of surfactant, which is necessary for both evolutionary and physiologic adaptation to atmospheric oxygen from fish to man. A wide variety of molecular insults disrupt such highly evolved physiologic cell-cell interactions, ranging from overdistention to oxidants, infection, and nicotine, all of which predictably cause loss of mesenchymal peroxisome-proliferator-activated receptor gamma (PPAR*γ*) expression and the transdifferentiation of lipofibroblasts to myofibroblasts, the signature cell type for lung fibrosis. By exploiting such deep cell-molecular functional homologies as targets for leveraging lung homeostasis, we have discovered that we can effectively prevent and/or reverse the deleterious effects of these pathogenic agents, demonstrating the utility of evolutionary biology for the prevention and treatment of chronic lung disease. By understanding mechanisms of health and disease as an evolutionary continuum rather than as dissociated processes, we can evolve predictive medicine.

## 1. Background

Normal lung development is the result of a functionally interconnected series of cell-molecular steps. This sequence of biologic events has been positively selected for evolutionarily over biologic time and space [1], resulting in optimal gas exchange mediated by alveolar homeostasis [2]. Elsewhere we have suggested that chronic lung disease (CLD) causes simplification of the lung in a manner consistent with the reversal of the evolutionary process [3, 4]. Therefore, by identifying those mechanisms that have evolved under selection pressure for optimal gas exchange [5], we have theorized that we can effectively reverse the deleterious effects of CLD by promoting the evolutionarily adaptive mechanism [6], rather than by just treating the symptoms [7]. By determining the cell-molecular sequence of spatiotemporal signals that have evolved the lung over phylogeny and ontogeny, we can identify physiologically rational targets for effectively preventing and reversing the deleterious effects of endogenous and exogenous factors known to irreversibly damage normal lung development and function.

The ground-breaking tissue culture experiments conducted by Grobstein in 1967 demonstrating that lung development was dependent on endodermal-mesenchymal interactions [8] led to decades of research to determine the underlying cell-molecular mechanisms. The seemingly simple epithelial-mesenchymal interactions during well-defined (embryonic, pseudoglandular, canalicular, saccular, and alveolar), but overlapping stages of lung development result in more than 40 different cell types [9]. Much of what we currently know about the mechanisms involved in lung development is derived from such studies of cultured lung cells signaling through growth factor-mediated pathways for proliferation and differentiation [10–12]. The discovery that epithelial-mesenchymal signaling induced the lipofibroblast via peroxisome proliferator-activated receptor gamma (PPAR*γ*) [13] gave rise to the hypothesis that normal lung development could be reconstituted [14] and recapitulated [15, 16]. The following recounts the essential role of PPAR*γ* in lipofibroblast differentiation and its exploitation for the effective treatment of the preterm lung.

## 2. Epithelial-Mesenchymal Interactions Generate Alveolar Lung Development

**Figure 1:**
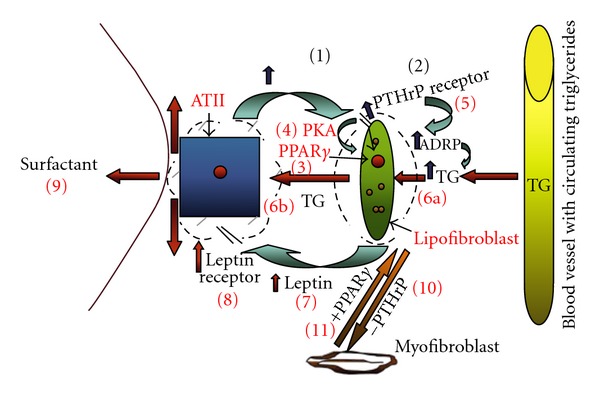
Schematic for paracrine determinants of alveolar homeostasis and disease.

The paracrine growth factor model used to study the maturation of the pulmonary surfactant system and the etiology of CLD is shown in the accompanying schematic (see Figure 1, *steps 1–11*). Briefly, we have observed coordinating effects of stretch on alveolar type II (ATII) cell expression of parathyroid-hormone-related protein (PTHrP) and PGE_2_ (Prostaglandin E_2_) (*step 1*), the lipofibroblast PTHrP receptor (*step 2*), PPAR*γ* upregulation (*step 4*) via Protein Kinase A activation (**step *3*), its downstream effect on lipofibroblast ADRP (Adipocyte-Differentiation-Related Protein) expression (*step 5*) and triglyceride (TG) uptake by both the lipofibroblast and the ATII cell (*steps 6a and 6b*), and on the interaction between lipofibroblast-produced leptin (**step *7*) and the ATII cell leptin receptor (**step *8*), stimulating de novo surfactant phospholipid synthesis by ATII cells (**step *9*). The schematic depicts lipofibroblast-to-myofibroblast transdifferentiation (**step *10*) due to decreased PTHrP following exposure to hyperoxia, volutrauma, or infection. All of these effects are shown to be prevented by PPAR*γ* agonists (**step *11*).

These studies were originally fostered by Barry Smith's seminal observation [10] that glucocorticoids accelerate ATII cell surfactant synthesis by stimulating fibroblast synthesis of an oligopeptide that he termed Fibroblast-Pneumonocyte Factor (FPF). It was known at that time that lung, prostate, and mammary mesodermal development were under endocrine control. Importantly, it was shown that early signals emanated from the epithelium to differentiate the immature mesenchyme in the neighboring epithelium of the developing mammary gland [17]. Moreover, Brody's laboratory had shown that the developing lung fibroblast acquired an adipocyte-like phenotype [18–20], termed the lipid-laden fibroblast, leaving open the question as to whether these cells might be a source of lipid substrate for surfactant synthesis by the ATII cell. The Torday laboratory later discovered the physiologic significance of these lipid-laden fibroblasts by coculturing them with type II cells, which resulted in the rapid trafficking of the lipid from the fibroblast to the type II cell, and its highly enriched incorporation into specific surfactant phospholipids. These data indicated the existence of a specific mechanism for the recruitment of lipid substrate from the vasculature to the type II cell for de novo surfactant synthesis. This trafficking was even more robust when the cocultured cells were treated with glucocorticoids, which are known to stimulate cell-cell interactions in the alveolus in association with increased surfactant synthesis, further reinforcing the notion of a putative mechanism for neutral lipid trafficking for surfactant synthesis since it appeared to be a regulated process [21].

Interestingly, the fibroblasts took up the neutral lipid, but did not release it unless they were in the presence of type II cells; conversely, the type II cells were unable to take up neutral lipid. These observations led to the discovery that type II cell secretion of prostaglandin E_2_ (Figure 1, *step 1*), a stretch- and glucocorticoid-regulated mechanism, caused the active release of neutral lipid from the fibroblasts [22]. This effect was further stimulated by glucocorticoid treatment of the lung fibroblasts [22], but the nature of the lipid uptake mechanism by the type II cells remained unknown. Yet we were well aware that the synthesis of pulmonary surfactant was a so-called “on demand” system [23–25], in which increased alveolar distension resulted in increased surfactant production, suggesting the existence of a stretch-sensitive signal emanating from the type II cell. With this in mind, we began studying the role of PTHrP in lung development because (a) it was expressed in the embryonic endoderm [26], (b) its receptor was present on the adepithelial mesoderm [27], (c) it had been shown to be stretch regulated in the urinary bladder [28] and uterus [29], and distension of the lung was known to be of physiologic importance in normal lung development [30], (d) knockout of PTHrP caused stage-specific inhibition of fetal lung alveolarization in the transition from the pseudoglandular to the canalicular stage [31].

Early functional studies of PTHrP had shown that it was a paracrine factor that stimulated surfactant phospholipid synthesis [32], and that it was stretch regulated [11] (Figure 1, *step 1*). We subsequently discovered that PTHrP stimulated neutral lipid uptake by developing lung fibroblasts (Figure 1, *steps 1 and 2*), which we chose to call lipofibroblasts [33], by upregulating ADRP (Figure 1, **step* 2*), a molecule previously shown to be necessary for lipid uptake and storage [34] (Figure 1, **step* 5*). We subsequently found that ADRP was the factor necessary for the uptake of neutral lipid by the lipofibroblast (Figure 1, **step* 6a*) and transit of neutral lipid from the lipofibroblast to the ATII cell for surfactant phospholipid synthesis (Figure 1, **step *6b*) [35, 36]. The missing component for the PTHrP regulation of lung surfactant was the putative lipofibroblast paracrine factor that empirically stimulated ATII cell surfactant synthesis (32). Reasoning that lipofibroblasts were homologs of adipocytes, we hypothesized that lipofibroblasts, like fat cells, expressed leptin, which would bind to the type II cell and stimulate surfactant synthesis—we found that lipofibroblasts did indeed express leptin during rat lung development, plateauing immediately prior to the onset of surfactant synthesis by the type II cell, and that leptin stimulates ATII cell surfactant synthesis [37] (Figure 1, *step 7*). Importantly, from a mechanistic standpoint, we discovered that type II cells express the leptin receptor [38] (Figure 1, *step 8*), thus providing a ligand-receptor signaling pathway between the lipofibroblast and type II cell. Moreover, PTHrP was discovered to stimulate leptin expression by fetal lung fibroblasts [37] (Figure 1, *steps 1, 2 and 7*), thus providing an integrated, growth factor-mediated homeostatic paracrine loop for the synthesis of pulmonary surfactant, as predicted by the PTHrP-based model of lung development.

Since the major inducers of bronchopulmonary dysplasia (BPD)—barotrauma [39], oxotrauma [40] and infection [41] —all cause ATII cell injury and damage, we investigated the effects of PTHrP deprivation on the lipofibroblast phenotype, only to discover that in the absence of PTHrP, the lipofibroblast transdifferentiates to a myofibroblast, the cell-type that characterizes lung fibrosis. Furthermore, myofibroblasts cannot support type II cell growth or differentiation, whereas lipofibroblasts can [13], demonstrating the functional significance of these two fibroblast phenotypes for lung development; importantly, when myofibroblasts are treated with a PPAR*γ* agonist, they revert back to the lipofibroblast phenotype, including their ability to promote type II cell growth and differentiation. As a result of these seminal observations, we have found that all of the above-mentioned BPD inducers cause downregulation of alveolar li-pofibroblast PPAR*γ* expression [38, 42, 43], inhibiting normal lung development. Moreover, in all of these conditions, PPAR*γ* agonists have been found to prevent delayed lung development, and in the case of nicotine inhibition of lung development, to even reverse this process [42–51].

## 3. The Evolution of Peroxisome Biology

Peroxisomes were first observed by Rhodin in 1954 [52] and were characterized as a novel cellular organelle by de Duve and Baudhin, whose laboratory first isolated peroxisomes from rat liver and determined their biochemical properties [53]. Since the core mechanisms involved in peroxisome biology are shared by a wide variety of organisms, it suggests a common evolutionary origin. Speculations about the evolution of peroxisomes began shortly after their discovery. Early photomicrographs suggested interactions between the peroxisome and endoplasmic reticulum (ER), leading some to speculate that peroxisomes were derived from the endomembrane system [54]. Subsequently, an alternative view that peroxisomes are independent organelles originating by endosymbiosis was proposed after it was observed that the peroxisomes formed from the division of existing peroxisomes, and that they import proteins [55], both features resembling those of bacterially derived organelles such as mitochondria and chloroplasts. But the most elaborate hypothesis regarding the evolutionary origins of the peroxisome was that of de Duve [56], who proposed a metabolic scenario for the establishment of an endosymbiosis mechanism that entailed the role of peroxisome enzymes in the detoxification of highly reactive oxygen species. In this scenario, the protoperoxisome was acquired at a time when the level of atmospheric oxygen was increasing and represented a toxic compound for the majority of living organisms. This concept is consistent with the evolution of the lung lipofibroblast [15] as an example of how vertebrates have entrained otherwise toxic substances in the environment as physiologic mechanisms [57]. Csete et al. [58] have observed that skeletal muscle satellite cells in culture will spontaneously become adipocytes in 21% oxygen, but not in 6% oxygen, suggesting that the episodic increases and falls in atmospheric oxygen over the last 500 million years may have caused the evolution of fat cells in the lung (lipofibroblasts) and periphery (adipocytes) [3]. Such a mechanism is a selection advantage since the lipofibroblast protects the alveolus against oxidant injury [59], and its production of leptin [37, 38] may have fostered modern-day stretch-regulation of alveolar surfactant [60–63], facilitating the increase in lung surface area [1, 4, 15] and mediating ventilation-perfusion matching [64]. The concomitant production of oxygen free radicals, lipid peroxides and other oxidative products likely generated eicosanoids (22) as a balancing selection for endogenous PPAR ligands. Bolstered by the popularity of the serial endosymbiotic theory [65], this view has been the most widely accepted among biologists.

More recently, the endosymbiosis theory for the origin of the peroxisome has been challenged. Experimental evidence shows a close relationship between the ER and peroxisome formation-certain peroxisomal membrane proteins must first be targeted to the ER before they reach the peroxisome [66], and peroxisome-less mutant yeast can form new peroxisomes from the ER upon introduction of the wild-type peroxisome gene [67]. And independent evidence for an evolutionary link between peroxisomes and the ER was provided by phylogenetic studies showing that homologous relationships between components of the peroxisomal import machinery and those of the ER-decay (ERAD) pathway [68, 69]. These data have led the research community to conclude that the peroxisome originates in the ER [70, 71], but have not excluded the possibility of an endosymbiont [71].

In the early 1990s,based on sequence homology with previously identified members of nuclear hormone receptor superfamily, three PPAR isotypes (PPAR*α*, *β*/*δ*, and *γ*) were identified, initially in Xenopus laevis and the mouse, and later in human, rat, fish, hamster, and chicken [72, 73]. These isotypes were initially shown to be activated by peroxisome proliferators, a group of substances able to induce peroxisome proliferation. Subsequently, various endogenous and exogenous PPAR ligands were identified, including fatty acids, eicosanoids, synthetic hypolipidemic, and antidiabetic agents [74]. Though PPARs are involved in several aspects of rodent development, they are most importantly involved in various aspects of lipid metabolism and energy homeostasis, with PPAR*γ*'s role in adipogenesis and lipid storage and PPAR*α*'s role in fatty acid catabolism in the liver being the best characterized [74, 75].

## 4. PPAR*γ* Mediates the Evolutionary History of the Adipocyte: Homologies Run Deep

Over the course of vertebrate evolution, during the Phanerozoic Period (the last 500 million years) the amount of oxygen in the atmosphere has increased to its current level of 21%. However, it did not increase linearly; instead, it increased and decreased several times, reaching concentrations as high as 35% and falling to as low as 15% over this time-period [76]. As pointed out above, the increased oxygen tension may have caused the differentiation of muscle satellite cells into lipofibroblasts, or lung adipocytes, in the lung, as the first directly affected anatomic site where the increased atmospheric oxygen would have generated selection pressure for evolutionary change. Consistent with this hypothesized adaptive response to the rising oxygen tension in the atmosphere, we have previously shown that the lipids stored in alveolar lipofibroblasts protect the lung against oxidant injury [59]. Like adipocytes, lipofibroblast differentiation requires upregulation of PPAR*γ* [13, 42, 44], which stimulates differentiation of myofibroblasts to lipofibroblasts [45]. In turn, the leptin secreted by the lipofibroblasts binds to its receptor on the alveolar epithelial cells lining the alveoli, stimulating surfactant synthesis [37, 38], and reducing alveolar surface tension. This results in a more deformable and efficient gas-exchange surface. Such positive selection pressure could have led to the stretch-regulated coregulation of surfactant and microvascular perfusion [77] by PTHrP, recognized physiologically as the mechanism of ventilation-perfusion matching. The evolution of these molecular mechanisms could ultimately have given rise to the definitive mammalian lung alveolus, with maximal gas exchange resulting from coordinate stretch-regulated surfactant production and alveolar capillary perfusion, thinner alveolar walls due to PTHrP's apoptotic or “programmed cell death” effect on fibroblasts [78], and a blood-gas barrier buttressed by type IV collagen [79]. We speculate that this last feature may have contributed generally to the molecular bauplan for the peripheral microvasculature of evolving vertebrates, given its effect on angiogenesis [80]. One physiologic consequence of the increased oxygenation may have been the concomitant induction of fat cells in the peripheral circulation, which led to endothermy or warm bloodedness- Mezentseva et al. [81] have shown that thermogenic fat cells differentiate from embryonic limb bud mesenchymal cells in association with the expression of PPAR*γ*. The resulting increase in body temperature synergized increased lung oxygenation because lung surfactant is 300% more active at 37°C than at ambient atmospheric temperature (i.e., the body temperature for cold-blooded organisms). For example, map turtles (Graptemys geographica) show different surfactant compositions depending on the ambient temperature [82]. Therefore, the advent of thermogenesis would have facilitated the physical increase in lung surfactant surface-tension-lowering activity. Moreover, it has been shown that treatment of cold blooded lizards with leptin, a product of adipocytes, increases their body temperature [83]. These synergistic selection pressures for adipogenesis would have been further functionally enhanced by the coordinate physiologic effects of epinephrine on the heart [84], lung [85], and fat depots [86], underpinned structurally by the increased production of leptin by fat cells, which is known to promote the formation of blood vessels [80] and bone [87], accommodating the infrastructural changes necessitated by the evolution of complex physiologic traits.

## 5. Everything Put Together Falls Apart in Bronchopulmonary Dysplasia

Since BPD can be induced by all of the varied factors cited above, disrupting epithelial-mesenchymal interactions, we designed experiments to determine the spatiotemporal effects of these disruptors on PTHrP-PPAR*γ* signaling. The effective distension of the newborn lung has a profound physiologic effect on pulmonary homeostasis [60, 61], and stretching of the ATII cell increases the expression and production of PTHrP [11]. In contrast, overdistension of the type II cell [88] results in downregulation of PTHrP expression, and hence PPAR*γ*, simulating the consequences of volutrauma [43]. Since hyperoxia also augments the transdifferentiation of lipofibroblasts to myofibroblasts *in vitro* [44], we determined the occurrence of hyperoxia-induced alveolar lipo-to-myofibroblast transdifferentiation *in vivo*. Either 24 hour or 7d *in vivo* exposure to hyperoxia significantly decreased the expression of lipogenic markers, and significantly increased the myogenic markers in association with arrested alveolarization; the lungs demonstrated relatively larger air spaces, thinned interstitia, decreased secondary septal crest formation, and a significant reduction in radial alveolar counts. Moreover, since lung inflammation is a key factor predisposing preterm infants to BPD, we determined the effects of lipopolysaccharide (LPS) on key alveolar epithelial-mesenchymal paracrine interactions [46]. There were acute (24 hour), significant increases in the expression of PTHrP, PPAR*γ*, ADRP, and surfactant protein-B (SP-B), without any significant effects on the expression of *α*-smooth muscle actin (*α*SMA). This was followed (72 h) by significant decreases in the expression of PTHrP, PPAR*γ*, ADRP, and SP-B, accompanied by a significant increase in the expression of *α*SMA, the key molecular and functional marker for BPD. And since nicotine affects lung growth and development [47], we determined the effect of *in utero* nicotine exposure on epithelial-mesenchymal interactions as well. Nicotine indirectly inhibited ATII cell proliferation and metabolism via its paracrine effects on the adepithelial lipofibroblasts [48], causing lipo-to-myofibroblast transdifferentiation [49, 89]. In all of the above-cited studies, a PPAR*γ* agonist blocked the disruptive effects, even reversing them in the case of nicotine.

## 6. PPAR*γ* Agonists Turn on a “Master Switch” for Normal Lung Development That Universally Prevents BPD

It is clear from the work outlined above that lipofibroblast PPAR*γ* signaling plays a central role in epithelial-mesenchymal interactions by maintaining alveolar homeostasis in volutrauma, oxotrauma, infection, and nicotine-mediated lung injury. The lipofibroblast expresses PPAR*γ* in response to PTHrP signaling from the ATII cell, resulting in both the direct protection of the mesoderm against oxidant injury [59], and protection against atelectasis by augmenting surfactant protein [37] and phospholipid [38] synthesis. Molecular injury to either the ATII cell or the lipofibroblast downregulates this molecular signaling pathway, causing myofibroblast transdifferentiation. And as indicated above, myofibroblasts cannot promote ATII cell proliferation and differentiation [13], leading to the failed alveolarization characteristic of BPD [50]. In contrast, lipofibroblasts support ATII cell proliferation and differentiation under the influence of factors implicated in the pathogenesis of BPD. This scenario is validated by a plethora of *in vitro* [13, 44–46, 51, 89, 90] and *in vivo* [42, 43, 48, 89] studies. Importantly, these studies show that PPAR*γ* agonists such as Prostaglandin J_2_ and rosiglitazone can prevent or reverse myofibroblast transdifferentiation, potentially preventing the inhibition of alveolarization in the developing lung, the hallmark of CLD of the newborn [13, 42, 45, 47–49, 51, 89, 90].

## 7. Conclusion

Using a basic cell biologic approach to elucidate the pathophysiology of BPD based on evolved cell-physiologic principles, we have determined the paracrine cell/molecular mechanism by which stretch coordinates epithelial-mesenchymal signaling, upregulating key genes for the induction of the prohomeostatic lipofibroblast phenotype—including PPAR*γ*, ADRP, and leptin—and the retrograde stimulation of ATII cell surfactant phospholipid and protein synthesis by the lipofibroblast product leptin. Each of these paracrine interactions requires cell-specific receptors on adjacent cells derived from the endoderm or mesoderm, respectively, that is, PTHrP receptors on the mesoderm and leptin receptors on the endoderm, to specifically mediate the signaling pathways within each cell type. More importantly, we have exploited the cell-specific molecular nature of this mechanism in order to effectively and comprehensively prevent and treat lung injuries that affect this signaling pathway. By identifying deep homologous mechanisms that have determined both the phylogeny and ontogeny of the lung, by using exogenous PPAR*γ* agonists we have been able to prevent and even reverse the effects of a wide variety of injurious agents affecting the epithelial-mesenchymal interactions that have evolved to determine the gas-exchange surface of the lung [1–5].
